# Histone Deacetylase 7‐Derived Peptides Play a Vital Role in Vascular Repair and Regeneration

**DOI:** 10.1002/advs.201800006

**Published:** 2018-06-25

**Authors:** Yiwa Pan, Junyao Yang, Yongzhen Wei, He Wang, Rongkuan Jiao, Ana Moraga, Zhongyi Zhang, Yanhua Hu, Deling Kong, Qingbo Xu, Lingfang Zeng, Qiang Zhao

**Affiliations:** ^1^ State key Laboratory of Medicinal Chemical Biology and Key Laboratory of Bioactive Materials (Ministry of Education) College of Life Sciences Nankai University Tianjin 300071 P. R. China; ^2^ Cardiovascular Division Faculty of Life Science and Medicine King's College London London SE5 9NU UK; ^3^ Jiangsu Center for the Collaboration and Innovation of Cancer Biotherapy Cancer Institute Xuzhou Medical University Xuzhou Jiangsu 221000 China

**Keywords:** HDAC7‐derived peptide, ischaemia disease, tissue‐engineered vascular grafts (TEVGs), vascular progenitor cells (VPCs)

## Abstract

Cardiovascular disease is a leading cause of morbidity and mortality globally. Accumulating evidence indicates that local resident stem/progenitor cells play an important role in vascular regeneration. Recently, it is demonstrated that a histone deacetylase 7‐derived 7‐amino acid peptide (7A, MHSPGAD) is critical in modulating the mobilization and orientated differentiation of these stem/progenitor cells. Here, its therapeutic efficacy in vascular repair and regeneration is evaluated. In vitro functional analyses reveal that the 7A peptide, in particular phosphorylated 7A (7Ap, MH[pSer]PGAD), could increase stem cell antigen‐1 positive (Sca1^+^) vascular progenitor cell (VPC) migration and differentiation toward an endothelial cell lineage. Furthermore, local delivery of 7A as well as 7Ap could enhance angiogenesis and ameliorate vascular injury in ischaemic tissues; these findings are confirmed in a femoral artery injury model and a hindlimb ischaemia model, respectively. Importantly, sustained delivery of 7A, especially 7Ap, from tissue‐engineered vascular grafts could attract Sca1^+^‐VPC cells into the grafts, contributing to endothelialization and intima/media formation in the vascular graft. These results suggest that this novel type of peptides has great translational potential in vascular regenerative medicine.

## 1 Introduction

Cardiovascular disease (CVD) represents a major cause of morbidity and mortality globally. Intimal injury is a common pathological basis of the process of CVD, and the recovery of injured endothelium is essential for protection against CVD.^1^, ^2^ Dysfunction of endothelial cells (ECs) is thought to play a pivotal role in injured endothelium, and EC health has been suggested to be maintained by a consistent supply of supporting growth factors and activation of local stem/progenitor cells that are capable of differentiation toward ECs.^3^, ^4^ It is well documented that vascular progenitor cells (VPCs), which are mainly located in the adventitia, contribute to the repair of the injured endothelium via migration toward the endothelium and differentiation into an EC lineage.^5^, ^6^ These VPCs are also an important source for the regeneration of tissue‐engineered vascular grafts.^7^, ^8^

Vascular bypass surgery has been successfully employed for the therapy of vascular diseases. Unfortunately, in the case of small‐diameter vascular grafts (diameter less than 6 mm), a high rate of restenosis limits the clinical use of commercially available products. The main reason for this is the poor regeneration of vascular tissue including slow and poor endothelialization.^9^, ^10^ In addition, there is a compelling need for novel strategies to revascularize ischaemic tissues, and critical limb ischaemia continues to be a severe health problem globally. In many cases, surgical or graft‐based procedures are not possible, necessitating the development of molecular or cell‐based therapies to promote angiogenesis.^11^, ^12^

Histone deacetylases (HDACs) are a family of enzymes that remove acetyl groups from N‐acetylated lysine residues on histones and are involved in gene transcriptional regulation through modulating chromatin structures. HDAC7, a member of the class II HDACs, is specifically expressed in the vascular endothelium and contributes to the maintenance of vascular integrity during early embryogenesis.^13^, ^14^, ^15^ Recently, we demonstrated that a 7‐amino acid (aa)‐peptide (7A, MHSPGAD) could be alternatively translated from a short open reading frame within the 5′‐terminal untranslated region of mouse *Hdac7* mRNA in stem cell antigen‐1 positive (Sca1^+^) VPCs in vitro and in vivo. The serine residue within the 7A peptide could be phosphorylated by the activated kinase MEKK1 S393, which in turn could transfer the phosphate group to the Thr145 site of 14‐3‐3γ, forming a novel MEKK1‐7A‐14‐3‐3γ signaling pathway downstream of vascular endothelial growth factor (VEGF). This novel signaling pathway contributed to the activation of the Sca1^+^‐VPCs.^16^ In the present study, we focused on the functional analysis of this 7A peptide and its translational potential in vascular repair and regeneration by using different disease and transplantation models.

## 2 Results

### 2.1 The 7A Peptide Increased VPC Migration and Differentiation toward the EC Lineage

Recently, we found that endogenous 7A peptide could only be detected in in vitro cultured Sca1^+^‐VPCs or Sca1^+^ cells in injured femoral arteries and not in Sca1^+^ cells in normal vessels. Therefore, it is very important to investigate the relationship between 7A expression and the activation of Sca1^+^ VPCs. Considering 7A can function as a phosphate carrier, a synthetic phosphorylated 7A (7Ap) (MH[pSer]PGAD) was included in the functional analyses. As controls, scramble 7S (MPHASGD) and 7Aa (MHAPGAD), in which the serine of the 7A is substituted by alanine to totally abolish phosphorylation, were also included in this study.

**Figure 1:**
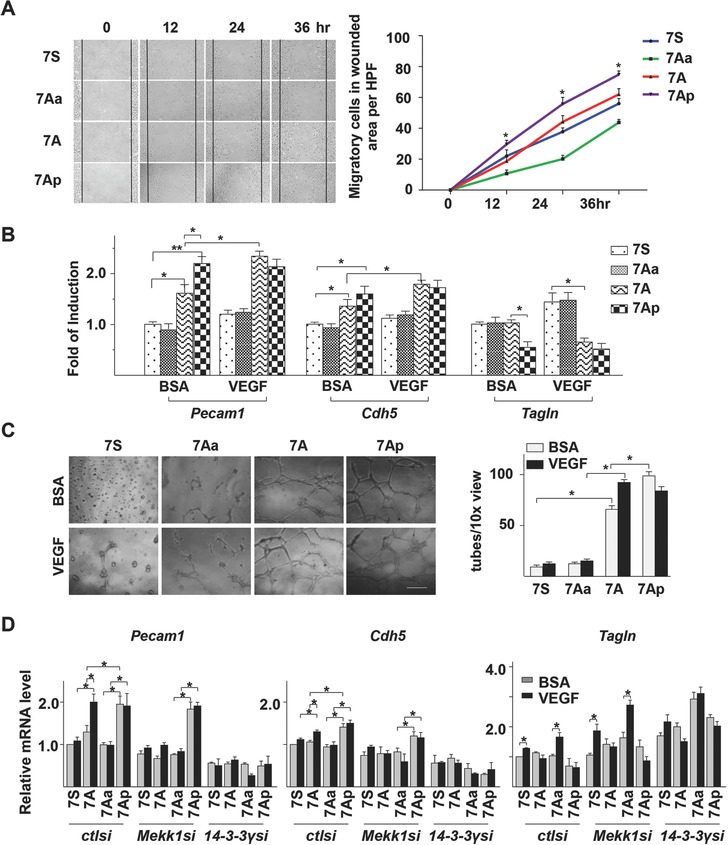
7A and 7Ap peptides increased migration and differentiation toward the EC lineage. A) 7Ap significantly increased VPC migration in a wound healing model. Wound was introduced into confluent VPCs by tip scratching, and incubated with DMEM medium containing 2% FBS and 1 ng mL^−1^ of 7S, 7Aa, 7A, or 7Ap peptide. Images were taken at 0, 12, 24, and 36 h postscratching (left, Scale bar: 100 µm). The migrated cells in scratched area were counted from three views per scratching, three scratchings per well, and three wells per peptide (right). Data presented are representative images or mean of three independent experiments. *: *p* < 0.05 (7Ap vs 7S) (*n* = 6, one‐way‐ANOVA followed by Tukey's post hoc analysis). B,C) 7A/7Ap increased VPC differentiation toward the EC lineage. The 3 d spontaneously differentiated VPCs were incubated with differentiation medium containing 1 ng mL^−1^ peptides and 10 ng mL^−1^ VEGF for 4 d, followed by quantitative RT‐PCR analysis with GAPDH as house‐keeping gene (B) or tube formation assay (C, Scale bar: 200 µm). 1% BSA was included as vehicle control. The data presented are representative images or mean of three independent experiments (*n* = 6, two‐way ANOVA followed by Dunnett's multiple comparison tests). D) MEKK1‐7A‐14‐3‐3γ mediated 7aa‐peptide‐induced VPC differentiation toward EC lineage. The VPCs were transfected with siRNA and cultured in differentiation medium for 3 d, followed by further differentiation with same protocol described above for 4 d, followed by quantitative RT‐PCR analysis of *Pecam1*, *Cdh5*, and *Tagln* mRNA levels with *Gapdh* as house‐keeping gene. 1% BSA was included as vehicle control. The data presented are representative images or mean of three independent experiments. *: *p* < 0.05. **: *p* < 0.01 (*n* = 6, two‐way ANOVA followed by Dunnett's multiple comparison tests).

As shown in **Figure** **1**A, 7A especially 7Ap accelerated Sca1^+^‐VPCs migration. As expected, 7Aa retarded Sca1^+^‐VPCs migration. Wound healing can be a combined effect of cell migration and proliferation. To assess whether 7A/7Ap‐increased migration was derived from the combined effect, a Br‐dU incorporation assay was performed. Under the serum‐free condition, the 7A peptide by itself could not support Sca1^+^‐VPC proliferation (Figure S1A, Supporting Information). Further experiments revealed that 7A had no effect on cell survival under oxidative stress (Figure S1B, Supporting Information).

The vessel wall resident Sca1^+^‐VPCs can differentiate toward both EC and smooth muscle cell (SMC) lineages.^17^, ^34^ When embryonic stem cells differentiate into SMCs, the *Hdac7* transcript variant 2 undergoes further splicing, leading to the incorporation of 7A into the far N‐terminal end of the HDAC7 protein.^18^ Thus, we assumed that 7A might be involved in the cell differentiation process. To test this, Sca1^+^‐VPCs were cultured in differentiation medium in the presence of 7‐aa peptides and/or VEGF, followed by quantitative RT‐PCR analysis of EC and SMC marker expression. As shown in Figure 1C, 7A increased *Pecam1* (CD31) and *Cdh5* (CD144) mRNA levels, which were significantly enhanced by VEGF, although VEGF alone had only a slight effect. The effect of 7Ap alone on *Pecam1* and *Cdh5* expression was comparable to the combined effect of 7A and VEGF (Figure 1B left and middle). 7A alone had no effect on *Tagln* (SM22) expression but significantly decreased *Tagln* expression in the presence of VEGF, although VEGF had a slight increasing effect. 7Ap alone significantly decreased *Tagln* expression. These results suggest that 7A, especially 7Ap, favors Sca1^+^‐VPC differentiation toward EC but not SMC lineages. The EC differentiation was further confirmed by tube formation assays (Figure 1C). Interestingly, although 7Ap alone was very effective in EC differentiation, the effect was somewhat decreased in the presence of VEGF (Figure 1B,C).

The effect of 7Ap on mature EC/SMC migration and proliferation was assessed with transwell migration and Br‐dU incorporation assays on human umbilical vein endothelial cells (HUVECs) and human vascular smooth muscle cells, respectively. The scramble 7Sp (MPHA[pS]GD) was included as a control. As shown in Figure S2A (Supporting Information), 7Ap increased Sca1^+^‐VPCs migration but had no effect on the migration of either ECs or SMCs. Furthermore, Br‐dU incorporation assays showed that 7Ap had no effect on cell proliferation of all three cell lines at a concentration of 1 ng mL^−1^ under serum‐free conditions (Figure S2B, Supporting Information).

To test whether 7A/7Ap‐mediated Sca1^+^‐VPCs differentiation toward EC was also through the novel MEKK1‐7A‐14‐3‐3γ signal pathway, *Mekk1* and *14‐3‐3γ* knockdown via siRNA transfection was introduced into Sca1^+^‐VPCs, followed by analyzing EC/SMC differentiation markers via quantitative RT‐PCR. As shown in Figure 1D, *Mekk1* knockdown abolished 7A/VEGF‐induced EC differentiation but had no effect on 7Ap‐induced EC differentiation, while *14‐3‐3γ* knockdown ablated both 7A/VEGF and 7Ap‐induced EC differentiation. These results suggest that the MEKK1‐7A‐14‐3‐3γ signal pathway is indeed involved in Sca1^+^‐VPCs differentiation toward EC lineage. Strikingly, knockdown of *Mekk1* especially *14‐3‐3γ* significantly increased *Tagln* expression (Figure 1D).

### 2.2 The 7aa‐Peptide Enhanced Vascular Injury Repair and Angiogenesis in Ischaemic Tissues In Vivo

**Figure 2:**
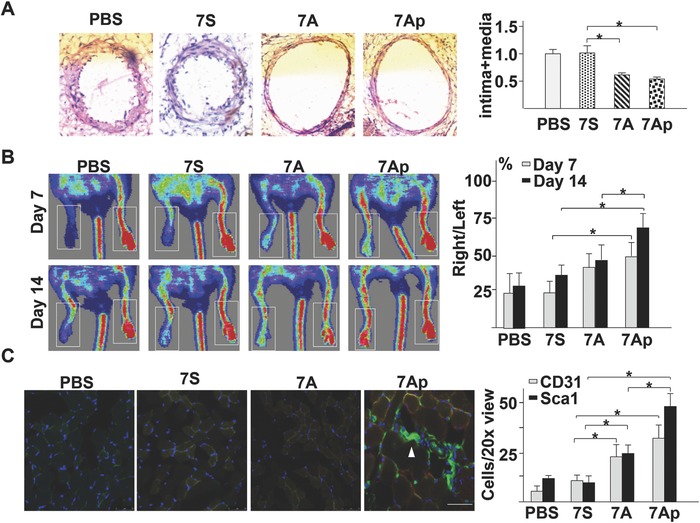
The peptide 7Ap increased vascular injury repair and angiogenesis in ischemic tissues in vivo. A) The peptides 7A and 7Ap attenuated neointima formation in a mouse femoral artery wire‐guided injury model. The left panel shows the H&E staining images of the injured vessel sections four weeks postsurgery. Scale bar: 100 µm. The right panel shows the average intima plus media area, with the value for the PBS group set as 1.0 (*n* = 6, one‐way‐ANOVA followed by Tukey's post hoc analysis). B) The peptide 7Ap increased foot blood perfusion in mice with a hindlimb ischemia model, which was introduced into six‐month‐old C57bl/6 mice, with 200 µL Pluronic‐127 gel containing 1 ng mL^−1^ peptides applied around the injured vessels. The left panel shows the Doppler Scanner images. The right panel shows the average ratio of blood flow in the right side (injured) to the left side (uninjured) (*n* = 6, two‐way ANOVA followed by Dunnett's multiple comparison tests). C) The peptide 7Ap increased Sca1^+^ (green) cell migration into the ischemic tissue and differentiation into CD31^+^ cells (red). DAPI was included to counterstain the nuclei. The left panel depicts representative immunofluorescence staining images on a skeletal muscle section from the injured leg. Arrow indicates the Sca1^+^ cell niche. The right panel shows the mean ± SEM CD31^+^ or Sca1 ^+^ cells from six 20× views. Scale bar: 20 µm. *: *p* < 0.05. **: *p* < 0.01 (*n* = 6, two‐way ANOVA followed by Dunnett's multiple comparison tests).

As described above 7A, especially 7Ap, could facilitate VPC mobilization and differentiation toward the EC lineage, suggesting that the 7A peptide may have therapeutic potential in vascular injury repair and angiogenesis in ischaemic tissues. To test this, the wire‐guided femoral artery injury model was created in ApoE^−/−^ mice.^19^ Pluronic‐127 gel containing 10 ng mL^−1^ of peptides or PBS was applied surrounding the adventitia of the injured vessels, which were harvested four weeks postsurgery. The hematoxylin and eosin (H&E) staining of cryo‐sections revealed that 7A or 7Ap administration significantly reduced neointima formation (**Figure** **2**A).

Furthermore, a hindlimb ischaemia model was introduced in eight‐month (Figure 2B) and ten‐week (Figure S3, Supporting Information) old C57BL/6J mice, in which Pluronic‐127 gel containing 10 ng mL ^−1^ peptides was applied surrounding the injured vessels.^20^ Foot blood perfusion was measured by a Doppler Scanner on day 7 and day 14 postsurgery. Young mice showed better foot blood perfusion recovery compared to aged mice (PBS group in Figure S3, Supporting Information, vs Figure 2B). Importantly, 7Ap significantly promoted recovery in both groups of mice, which might be due to the increased formation of a new Sca1^+^ cell niche in the ischaemic tissues (Figure 2C).

### 2.3 Fabrication of Peptide‐Loaded Tissue Engineered Vascular Grafts (TEVGs)

**Figure 3:**
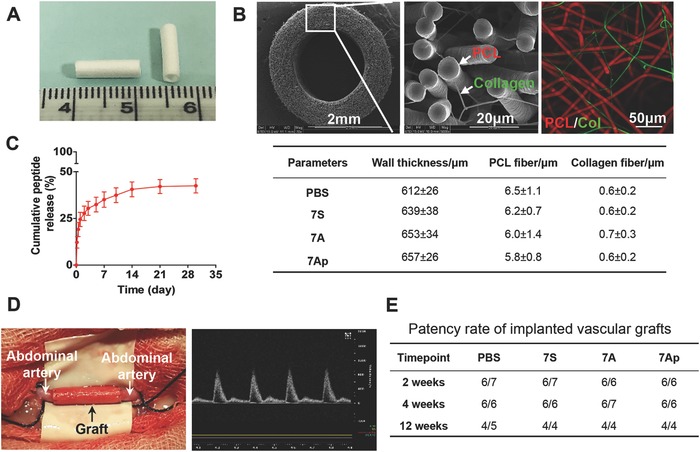
The fabrication of the peptide‐loaded tissue engineered vascular grafts. A) The images show the morphology of the tubular vascular grafts, the microstructure as revealed by scanning electronic microscope, and B) (upper panel) the distribution of the two types of fibers of PCL revealed as red by DiI and collagen revealed as green by DiO. B) (lower panel) The graft parameters are summarized. C) In vitro release of 7A peptide from the TEVG (*n* = 3). D) Implantation of the graft in a rat to replace the abdominal artery (left), and the ultrasound scanning image of the implanted TEVGs (right). E) The patency rate of implanted TEVGs at different timepoints.

To evaluate whether the 7A peptide has therapeutic potential, TEVGs were fabricated by a co‐electrospinning technique.^9^ The TEVGs were sized at 2.0 mm diameter and 600 µm wall thickness (**Figure** **3**A). They showed a micro/nanohybrid fibrous structure, consisting of PCL microfibers (≈6 µm) to provide mechanical support and structural maintenance and collagen nanofibers (≈600 nm) to act as a carrier for delivery of peptides (Figure 3B). The loading amount is about 417.5 ng for the graft of 1 cm length that has been calculated from the feeding ratio of electrospinning. Both fibers were distributed homogeneously across the graft wall as revealed by scanning electronic microscopy (SEM) (Figure 3B middle). The distribution of the two types of fibers could also be clearly identified by double labeling showing PCL in red and collagen in green (Figure 3B right). The peptides (7S, 7A, 7Ap) released from the grafts as a result of the degradation of the collagen fibers exhibited a sustained profile over a period of 30 d. The cumulative release reached ≈43 ± 7% (s.d.) of the theoretical value (Figure 3C) in 7A group, and a similar releasing profile has been observed in 7S and 7Ap groups (Figure S4, Supporting Information). The TEVGs (1.0 cm in length) were further evaluated by in vivo implantation in a rat abdominal artery replacement model (Figure 3D left). The patency of the implanted grafts was first examined by ultrasound at different timepoints (Figure 3D, right). Most of the grafts had good patency without aneurysms or bleeding and patency rates were higher in the 7A/7Ap groups than in the PBS and 7S groups (Figure 3E).^21^, ^38^ Further analysis by stereomicroscopy revealed that the luminal surfaces of all groups were smooth, clean and free of thrombi at 12 weeks (Figure S5, Supporting Information).

### 2.4 7Ap Promoted Vascular Regeneration and Remodeling in TEVGs

**Figure 4:**
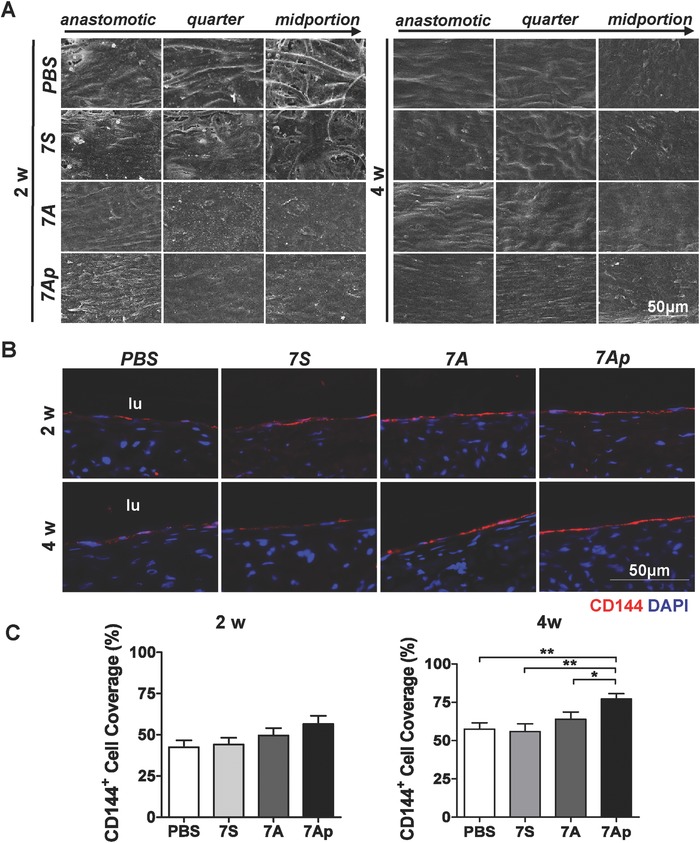
7Ap effectively increased endothelialization of the TEVGs. The TEVGs were implanted into rats to replace the abdominal artery and collected at two and four weeks postimplantation, followed by observation of the endothelialization on the lumen side of the TEVGs using A) scanning electronic microscope or B) CD144 staining of the sections. C) The endothelialized coverage was calculated by the CD144 positive cells covered area as a percentage of the entire lumen area. DAPI was used to counterstain the nuclei. Data are represented as the mean ± SEM for each group. *: *p* < 0.05. **: *p* < 0.01 (*n* = 6, one‐way‐ANOVA followed by Tukey's post hoc analysis).

The endothelialization on the lumen side of the implanted TEVGs was first observed by scanning electronic microscopy at three locations selected continuously from the anastomotic site to the midportion. As shown in **Figure** **4**A, the lumen side of the 7A and 7Ap‐loaded TEVGs was already covered by a layer of cells at two weeks postimplantation. In contrast, the PBS and 7S‐loaded TEVGs were only partially covered, and bare fibers could still be identified in the midportion. After four weeks postimplantation, all of the grafts were almost fully covered by a confluent layer of neo‐tissue. In the 7A group, especially the 7Ap group, the cells oriented along the blood flow, resembling the endothelium of the native artery. 4′,6‐diamidino‐2‐phenylindole (DAPI) staining revealed that a number of cells had incorporated into the wall of all of the TEVGs (Figure 4B). Immunofluorescence staining demonstrated that there was a layer of ECs on the lumen side (Figure 4B). The statistical analysis of the CD144^+^ cell‐covering area via immunofluorescence staining on the longitudinal sections revealed that 7Ap‐loaded TEVGs had much higher endothelialization rate compared to other groups (Figure 4C and Figures S6 and S7, Supporting Information). After 12 weeks postimplantation, all of the grafts were almost fully endothelialized without detectable differences (Figure S8, Supporting Information). These results suggest that 7Ap can facilitate endothelialization of TEVGs.

**Figure 5:**
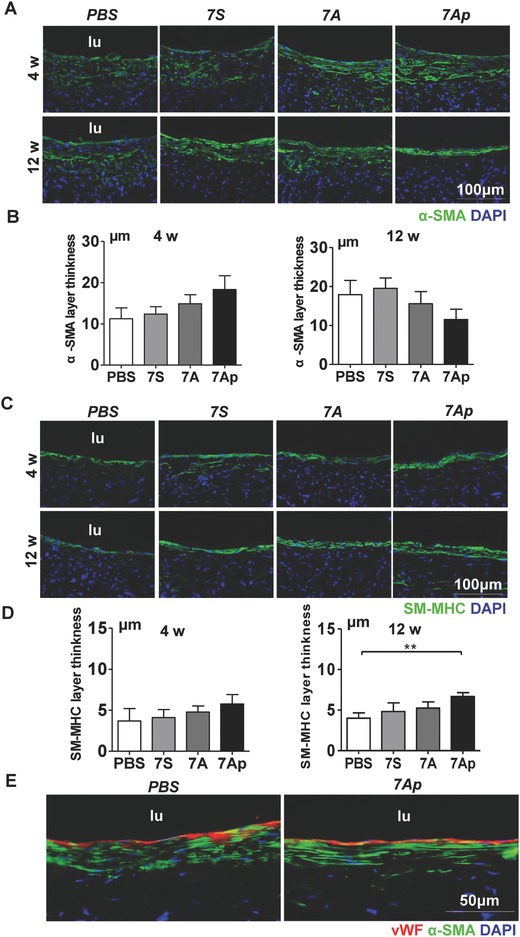
7Ap increased EC‐SMC interactions in the implanted TEVGs. Immunofluorescence staining was performed on TEVGs sections to observe the recruitment of SMCs by using A) anti‐α‐SMA and B) anti‐MHC, and quantification of the average layer thickness of B) α‐SMA and D) SM‐MHC positive cells. E) The interaction between ECs and SMCs was observed by double immunofluorescence staining with anti‐α‐SMA (green) and anti‐vWF (red). DAPI was included to counterstain the nuclei. Data are represented as the mean ± SEM for each group (*n* = 6 for 4 weeks, and *n* = 4 for 12 weeks). **: *p* < 0.01 (one‐way‐ANOVA followed by Tukey's post hoc analysis).

In the native arterial vessels, ECs form a thin layer of intima while one to several layers of SMCs lie in the media. To assess whether the endothelialization of the TEVGs was accompanied by SMC layer formation, immunofluorescence staining was first performed on the cross sections with anti‐α‐SMA antibody. As shown in **Figure** **5**A, a layer of SMA^+^ cells could be observed after four weeks, and the SMCs were well organized. The evolution of α‐SMA^+^ layers from 4 to 12 weeks showed a divergent tendency among the four groups, that is, their thickness decreased in the 7A and 7Ap groups and increased in the PBS and 7S groups (Figure 5B) . This indicates that 7A, especially 7Ap, could reduce the overproliferation of SMCs that lead to adverse intimal hyperplasia.

The regeneration of mature smooth muscle (SM‐MHC^+^) did not show any significant differences at four weeks postimplantation (Figure 5C). After 12 weeks, the thickness of the SM‐MHC^+^ layer was remarkably higher in the 7Ap group than in the PBS one (Figure 5D), suggesting that 7Ap could modulate the infiltrated SMCs into a more mature contractile phenotype. Furthermore, double immunofluorescence staining with anti‐vWF (red) and anti‐α‐SMA (green) antibodies exhibited better tissue regeneration in the 7Ap group compared to the control group, showing a layer of neo‐endothelium and several layers of aligned SMCs beneath (Figure 5E). At the same time, we noticed that some of the vWF‐positive cells were SMA‐positive as well. To address whether there is endothelial‐to‐mesenchymal transition, further detailed investigation is required.

Successful vascular grafts need capillary vessels to support cells in the graft wall. Immunofluorescence staining with anti‐CD31 antibody on the cross sections of the TEVGs revealed that the addition of 7A especially 7Ap effectively increased capillary vessel formation even at two weeks postimplantation (Figure S9A, Supporting Information). After four weeks, the density of capillary vessels in 7Ap group was significantly higher than that in other groups (Figure S9B, Supporting Information). These results suggest that 7A especially 7Ap can increase capillary vessel formation. The 7Ap‐loaded TEVGs resembled native vasculature more closely.

### 2.5 7Ap Stimulated Local Vascular Stem/Progenitor Cells Migration and Differentiation into the EC Lineage

**Figure 6:**
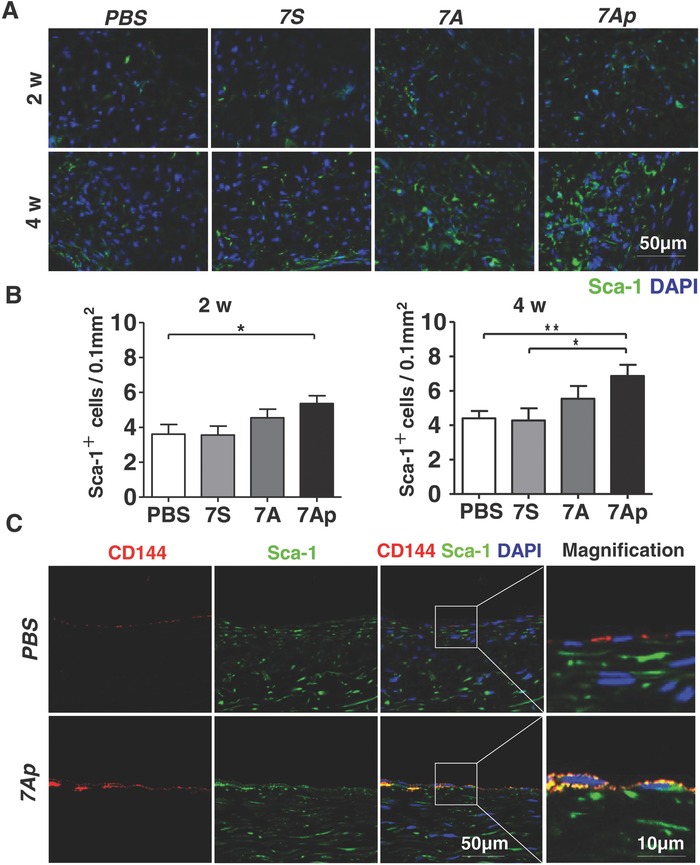
7Ap increased Sca1^+^‐VPC differentiation toward ECs contributing to endothelialization of TEVGs. A) Immunofluorescence staining was performed to detect the recruitment of Sca1^+^‐VPCs using anti‐Sca1 antibody on the implanted TEVGs sections at time indicated, and B) quantification of the infiltrated Sca1^+^ cells within 0–100 µm depth from the lumen by randomly selecting six views (40×) from each cross section. C) The contribution of the Sca1^+^ progenitor cells‐derived ECs to the endothelialization was detected by double immunofluorescence staining with anti‐Sca1 (green) and anti‐CD144 (red) antibodies on the four weeks implanted TEVGs sections. DAPI was used to counterstain the nuclei. Data are represented as the mean ± SEM for each group (*n* = 6 at 2 and 4 weeks, and *n* = 4 at 12 weeks). *: *p* < 0.05; **: *p* < 0.01 (one‐way‐ANOVA followed by Tukey's post hoc analysis).

As our in vitro studies have shown that 7A, especially 7Ap, could increase Sca1^+^‐VPC migration and differentiation toward the EC lineage, we wondered whether the local VPCs contributed to the neo‐vasculature formation in the TEVGs. To test this, immunofluorescence staining was performed with anti‐Sca1 antibody on sections from two‐ and four‐week implanted TEVGs. As shown in **Figure** **6**A, the infiltration of Sca1^+^‐VPCs within the graft walls could be detected at two weeks postimplantation. As time went on, the Sca1^+^ cell number increased. Very importantly, 7A, especially 7Ap, enhanced this process, since the 7Ap group had the highest number of Sca1^+^ cells (Figure 6B). Further experiments with double immunofluorescence staining using CD144 (red) and Sca1 (green) antibodies (Figure 6C) revealed that the majority of the CD144^+^ cells were also Sca1‐positive (Figure 6C, magnified image), indicating that these new ECs are derived from the Sca1^+^‐VPC differentiation.

**Figure 7:**
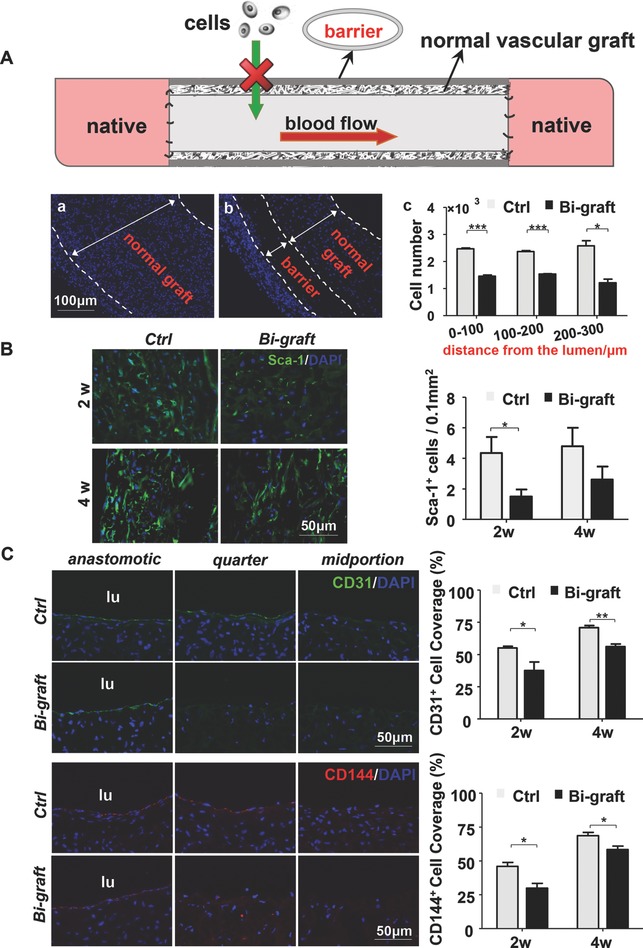
Bi‐layered PCL TEVGs revealed that the Sca1^+^‐VPCs were mainly derived from the surrounding tissues. A) An illustration of the bi‐layered PCL fabricated TEVGs. B,C) The outer layer with small pores reduced Sca1^+^‐VPCs recruitment into the TEVGs wall and the lumen endothelialization of the TEVGs. B) The single PCL and bi‐layered PCL fabricated TEVGs were implanted into rats to replace the abdominal arteries, and harvested at two and four weeks, respectively, postimplantation, followed by immunofluorescence staining with anti‐Sca1 antibody to show the recruitment of Sca1^+^‐VPCs in the TEVGs wall or C) with anti‐CD31 (green, upper) and anti‐CD144 (red, lower) antibodies to show the lumen endothelialization. DAPI was used to counterstain the nuclei. Data are represented as the mean ± SEM. *: *p* < 0.05; **: *p* < 0.01; ***: *p* < 0.001 (*n* = 6, two‐way ANOVA followed by Dunnett's multiple comparison tests).

It has been established that both local resident and circulating stem/progenitor cells contribute to vascular remodeling following vascular injury.^22^, ^23^, ^24^ To explore the origin of the infiltrated Sca1^+^cells and their contribution to the vascular remodeling in TEVGs, a bi‐layered vascular graft was employed in this study (**Figure** **7**A, upper panel). In addition to the regular vascular grafts that act as an inner layer, an external layer composed of PCL nanofibers was introduced as an outside barrier (Figure S10, Supporting Information).^25^ Due to its low porosity, this external layer could effectively restrict the infiltration of cells from the surrounding tissues after implantation for two weeks (Figure 7Aa,b).^26^ Quantitative analysis based on DAPI staining showed that the average cell number was significantly decreased within the graft wall, especially in the area near the lumen (Figure 7Ac). The infiltration of Sca1^+^‐VPCs was further compared between the two groups, and statistical data showed that the density of the Sca1^+^cells was markedly reduced due to the outside barrier at both two and four weeks (Figure 7B). All of these results confirm that surrounding tissues are an important source for the Sca1^+^‐VPCs infiltration within the vascular grafts.

Endothelialization of the two types of vascular grafts was compared by SEM observation (Figure S11, Supporting Information) and immunofluorescence staining (Figure 7C, Figures S12 and S13, Supporting Information). The results indicated that the outside barrier slowed down the endothelialization process, and the average coverage ratio of neo‐endothelium was significantly lower in the outside barrier (bi‐graft) graft than in the control one (Figure 7C). These results suggest that both the adjacent ECs^21^, ^27^ and the circulating and local resident stem/progenitor cells migrate into and contribute to vascular remodeling in the TEVGs.

**Figure 8:**
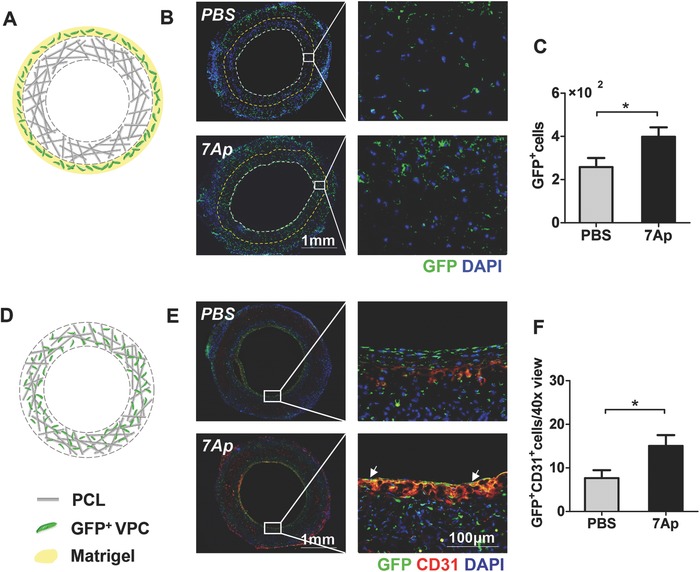
7Ap increased in vivo migration and differentiation of Sca1^+^‐VPCs preseeded in the TEVGs. A) Matrigel containing GFP‐Sca1^+^‐VPCs was applied surrounding the adventitia of the vascular grafts. B) Immunofluorescence staining was performed to detect the migration of seeded VPCs within 0–200 µm depth from the lumen (dotted area) after 3 d postimplantation, and C) the corresponding quantification on GFP‐Sca1^+^‐VPC. D) GFP‐Sca1^+^‐VPCs were seeded in the graft wall directly. E) Double immunofluorescence staining was performed to detect the differentiation of seeded VPCs after 7 d postimplantation using anti‐GFP (green) and anti‐CD31 (red) antibodies, and the corresponding quantification on GFP^+^CD31^+^VPC on the lumen side from five randomly selected views of each cross section. F) DAPI was included to counterstain the nuclei. Data are represented as mean ± SEM, *: *p* < 0.05 (*n* = 5, two‐tailed unpaired Student's *t*‐test).

To further verify the effect of 7Ap peptides on the migration of Sca1^+^‐VPCs from surrounding tissues into the grafts, Matrigel containing GFP‐Sca1^+^‐VPCs was applied surrounding the adventitia of vascular grafts (**Figure** **8**A). Immunofluorescence images of the cross sections showed that after 3 d postimplantation the number of cells migrated to the graft wall was significantly (*p* < 0.05) lower in the control group compared to the 7Ap group (Figure 8B,C). The fate of infiltrated Sca1^+^‐VPCs from surrounding tissues has also been investigated by seeding the GFP‐Sca1^+^‐VPCs into the graft wall directly (Figure 8D). Double immunofluorescence staining using CD144 and Sca1 antibodies demonstrated after 7 d postimplantation some Sca1^+^‐VPCs differentiated into ECs, and 7Ap significantly (*p* < 0.05) enhanced the number of double‐positive cells on the lumen side compared to the control group (Figure 8E,F). All of these results confirmed that Sca1^+^‐VPCs from the surrounding tissues can migrate transmurally and differentiate into the EC lineage in the TEVGs.

## 3 Discussion

Tissue‐engineered vascular grafts prepared by traditional strategies are time‐consuming and costly, which often restricts their clinical application. Instead, a new in situ (cell‐free) concept has received increasing attention, that is, a neo‐artery is completely generated in situ by taking advantage of the regenerative potential of the body.^28^ To stimulate or maximize this in situ regeneration capability, vascular grafts have been engineered in terms of physical structure^25^ and surface chemistry,^29^, ^30^ as well as the delivery of vasoactive molecules.^31^ In this study, we demonstrated that an *Hdac7‐*derived 7‐aa peptide can serve as a vasoactive molecule to mobilize local resident progenitor cells, contributing to in situ neo‐artery formation.

The origin of cells participating in the regeneration of vascular tissues remains a major obstacle restricting the success of in situ tissue engineering strategies. We tend to believe that vascular cells (ECs and SMCs) migrate from the anastomotic native tissues (transanastomotic ingrowth). Breuer and co‐workers have reported that the adjacent vessel wall is the principal source of these endothelial and smooth muscle cells when implanted a polymer‐based graft as an inferior vena cava interposition in mice.^27^ However, the results are often limited by many factors, including graft structure, implant site, and animal species. For example, cell migration from other directions (such as surrounding tissues) has been restricted due to the dense structure of the graft walls. It has been accepted that endothelial cell migration from the anastomotic sites is generally limited to 1 cm,^32^ which is far less than the clinical requirement of re‐endothelialization of relatively longer vascular grafts.^33^ Therefore, there is a need for recruitment of cells (both mature ECs and stem/progenitor cells) from sites beyond the anastomotic border via circulation or through migration from the surrounding tissue.

Vascular tissue‐resident stem cells have been discovered to display the capacity to differentiate into vascular cell lineages, which may contribute to the regenerative process.^34^ More specifically, the adventitial resident VPCs play a major role in the repair of the injured endothelium via migration toward the endothelium and differentiation into an EC lineage.^5^, ^6^

HDAC7 plays an important role in the maintenance of endothelium integrity.^35^, ^36^, ^37^ In a parallel study, we have demonstrated that mouse *Hdac7* mRNA can undergo alternative translation to produce a biological active 7‐aa peptide, which functions as a phosphate transfer carrier in cellular signal transduction. In this study, we have evaluated its application potential in peptide‐loaded tissue engineered vascular grafts by incorporating the 7A/7Ap peptides into the micro/nanofibrous vascular grafts.

In vitro studies demonstrated the 7Ap peptide is very powerful in Sca1^+^‐VPCs‐derived EC differentiation. However, we also noticed that 7Ap seemed less effective during EC differentiation in the presence of VEGF compared to 7Ap alone. This phenomenon may be due to the diverse functions of VEGF. In in vitro differentiation process, the increased EC cells can be derived from both the newly differentiated ECs and the proliferation of the differentiated mature ECs. When mature ECs undergo proliferation, the EC marker especially those involved in cell‐to‐cell contact such as CD31 (*pecam1*) and CD144 (*Cdh5*) will be downregulated to relieve the connection among adjacent cells. This will result in an observable decrease in EC marker expression at mRNA levels and tube formation. Although 7Ap had no effect on EC proliferation, VEGF is a mitogenic factor for EC. Thus, the combination of 7Ap and VEGF may show a decrease in EC marker expression and tube formation compared to 7Ap alone.

An intact endothelial cell monolayer has proven to be an antithrombogenic interface to maintain the patency of the implanted vascular grafts.^38^ In the meantime, the recruitment of SMCs is vital to stabilize the newly formed endothelium.^32^, ^39^, ^40^, ^41^, ^42^ In this study, we have demonstrated that the inclusion of 7A, especially the phosphorylated version 7Ap, significantly increased endothelialization. The majority of the ECs, especially those in the midportion of the TEVGs, may be derived from the local resident Sca1^+^ progenitor cells of the surrounding tissues, because 7Ap can serve as a chemoattractant for Sca1^+^‐VPCs and direct their differentiation toward the EC lineage. The ECs on the lumen of TEVGs will secrete relevant cytokines (such as PDGF or TGF‐β1) to recruit SMCs to stabilize the newly formed endothelium.^40^ The interactions between ECs and SMCs are very important for the function and homeostasis of the regenerated neo‐artery. Considering that 7Ap suppresses Sca1^+^‐VPCs differentiation toward SMCs and has no effect on SMC migration and proliferation, the recruitment of SMCs may be mainly mediated by ECs on the lumen of the TEVGs. After all, precise lineage tracing may be required to verify the contribution of Sca1^+^‐VPCs.

Therefore, 7Ap may help with the organization and maturation of SMCs within the TEVGs, which could also explain the discrepancy between in vivo SMC regeneration and in vitro SMC differentiation. Importantly, the Sca1^+^ progenitor cells also contribute to capillary vessel formation in the TEVGs wall, which is critical for the functional maintenance of the neo‐artery following scaffold degradation.

The dose‐dependency of the 7A/7Ap peptide is an important factor affecting therapeutic efficacy. 7A/7Ap was effective in EC differentiation at a concentration of 0.1 ng mL^−1^. For cell migration, it was more effective at a relatively higher concentration, and therefore 1 ng mL^−1^ was used. In in vivo studies, we assumed that there would be a concentration gradient extending from the carrier (F‐127 gel or collagen fiber), and the final concentration within the ischaemic tissue reached ≈0.1 ng mL^−1^. For future studies, it will be necessary to detect whether there is dose dependency.

In summary, the clinical potential of histone deacetylase 7 (HDAC7)‐derived 7‐amino acid peptides in vascular repair and regeneration has been systematically evaluated. The effect of the 7A peptide, especially the phosphorylated 7A peptide (7Ap), on VPC migration and EC differentiation has been investigated by in vitro functional analyses. Local delivery of 7A/7Ap increased re‐endothelialization and suppressed neointima formation in the femoral artery injury model, and promoted foot blood perfusion recovery in the hindlimb ischaemia model. Degradable tissue‐engineered vascular grafts loaded with peptides have been successfully prepared and evaluated in an abdominal aorta replacement model of rats. Sustained delivery of 7A, especially 7Ap, from tissue‐engineered vascular grafts enhanced endothelialization and intima/media formation in the vascular graft via inducing Sca1^+^‐VPC migration and differentiation. All of these results suggest that 7A/7Ap provides a promising therapeutic strategy for ischaemia diseases and vascular graft bypass surgery. Because the human orthologue of Sca1 has not been identified yet, it will be necessary to investigate whether 7A/7Ap has similar effect on human adult stem/progenitor cells, which will provide direct evidence for its possible application in translational medicine.

## Conflict of Interest

The authors declare no conflict of interest.

## Supporting information

Supplementary
