# Supplementary material for: Histone Deacetylase 7‐Derived Peptides Play a Vital Role in Vascular Repair and Regeneration
Source: Adv Sci (Weinh). 2018 Jun 25;5(8):1800006. doi: 10.1002/advs.201800006 (PMC6097091; doi:10.1002/advs.201800006)
Supplement: Supplementary file 1 — Supplementary [file ADVS-5-1800006-s001.pdf]

Copyright WILEY-VCH Verlag GmbH & Co. KGaA, 69469 Weinheim, Germany, 2018.

ADVANCED SCIENCE
Open Access

Supporting Information

for *Adv. Sci.*, DOI: 10.1002/advs.201800006

Histone Deacetylase 7-Derived Peptides Play a Vital Role in Vascular Repair and Regeneration

Yiwa Pan, Junyao Yang, Yongzhen Wei, He Wang, Rongkuan Jiao, Ana Moraga, Zhongyi Zhang, Yanhua Hu, Deling Kong, Qingbo Xu, Lingfang Zeng, and Qiang Zhao**

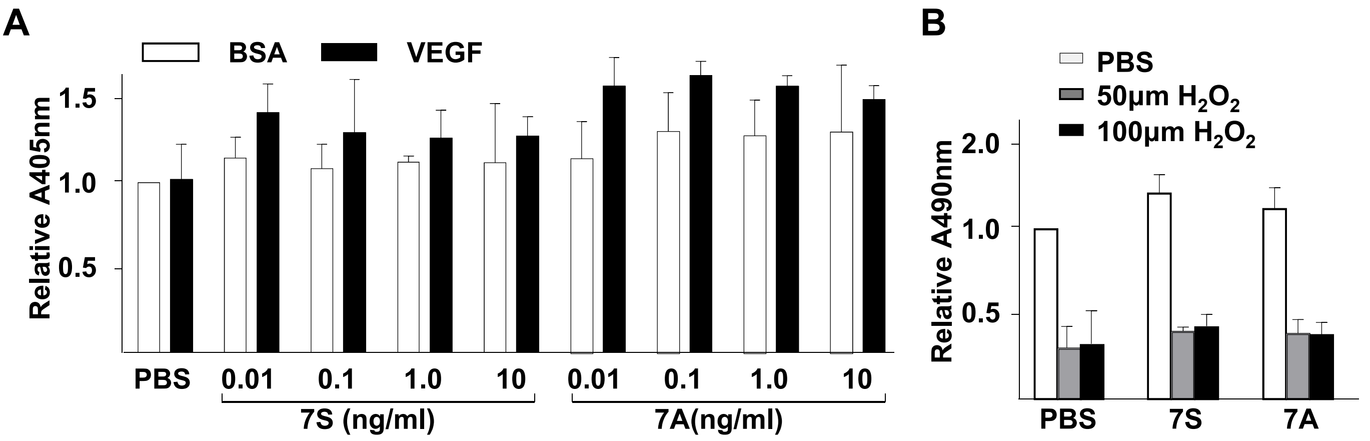

Figure S1. (A) The 7A peptide had no effect on VPC proliferation in serum free medium. VPCs were cultured in serum free DMEM containing different concentration of peptides with/without 5ng/ml VEGF as indicated for 24hr, and BrdU labelling reagent were added 6hr prior to harvesting the cells. PBS and 1%BSA were used as control for peptide and VEGF respectively. The relative A405nm was defined as that of PBS/BSA group set as 1.0. **(B) The 7aa-peptide had no effect on cell survival under oxidative stress.** The VPCs were treated with 50µm and 100µm H_2O_2 in the presence of 1ng/ml 7S or 7A peptide in serum free medium for 24hr, followed by MTT assays. The relative A490nm was defined as that of PBS group set as 1.0.

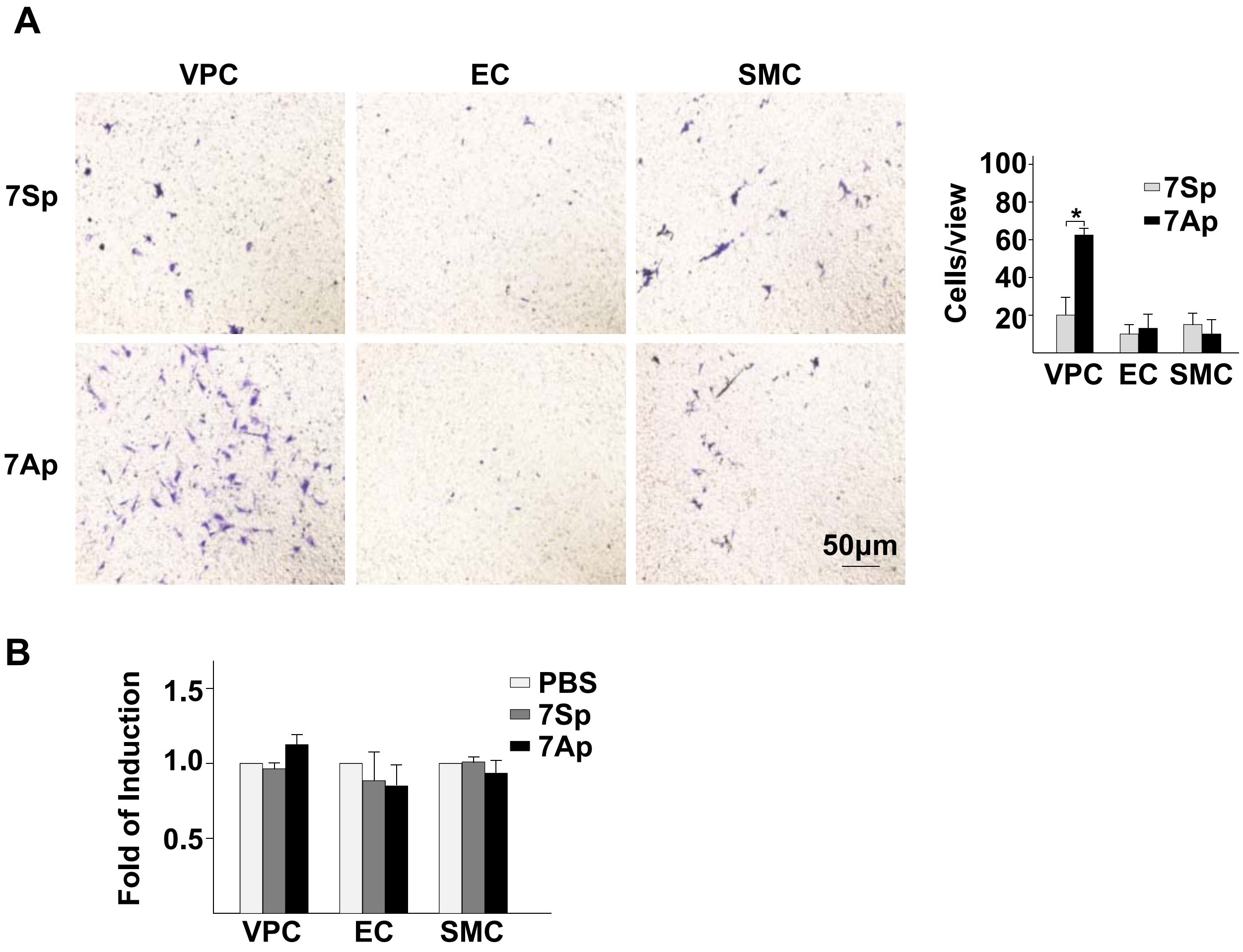

Figure S2. 7Ap increased VPC migration but had no effect on ECs and SMCs. (A) 7Ap increased VPC migration as measured by transwell migration assay. 3x104 cell/well of VPCs, HUVECs (EC) or HSMCs (SMC) were seeded in the insert of transwell with the holder filled with medium containing 0.5% FBS and 1ng/ml 7Sp or 7Ap. Migrated cells were assessed at 6hr post-seeding. Left panel shows the representative images, and right panel shows the quantitative analysis of migrated cells per 10x lenses view from 6x3 views. $p<0.05$. **(B)** 7Ap had no effect on VPC, EC or SMC proliferation under serum-free conditions. VPCs or ECs or SMCs were pre-treated with serum-free medium for 2hr and then treated with 1ng/ml 7SP or 7Ap in the presence of Br-dU for 6hr, followed by Br-dU incorporation assay. PBS was included as control. Fold of induction was defined as the ratio of A405nm with that of PBS group set as 1.0. Data presented are mean of three independent experiments.

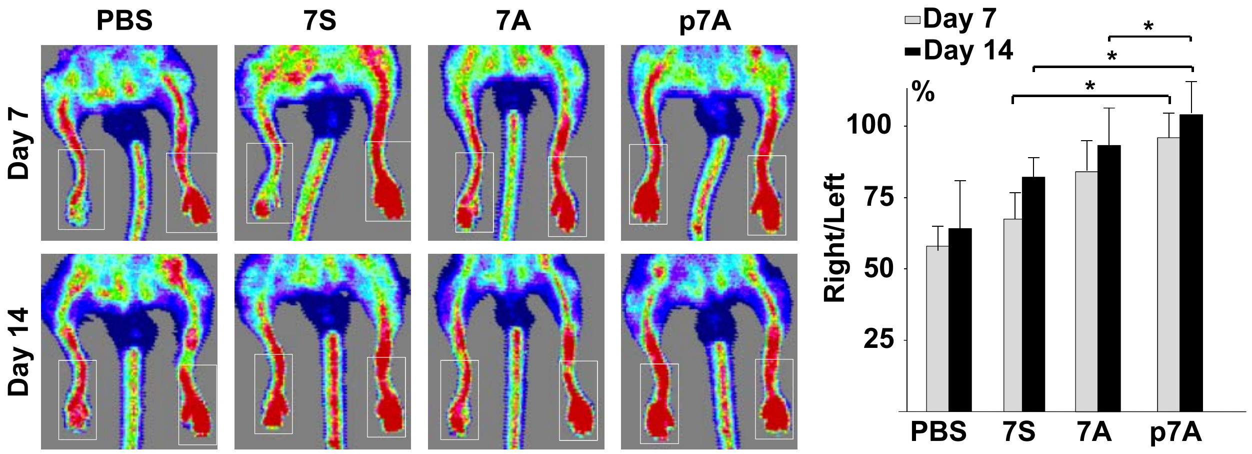

Figure S3. 7Ap significantly increased foot blood perfusion in hindlimb ischemia model. The hindlimb ischemia model was introduced into 10-week old C57bl/6 mice by ligating and cutting the femoral artery in the right side. 200μl of pluronic-F127 gel containing 1ng/ml peptides was applied to the injured area. Foot blood flow was measured by Doppler Scanner at day 7 and day 14 post-surgery. Left panel shows the representative images of 6 mice for each group. The right panel shows the ratio of foot blood flow in the right injured side to that of the left uninjured side. Scale bar: 50μm. *: $p<0.05$.

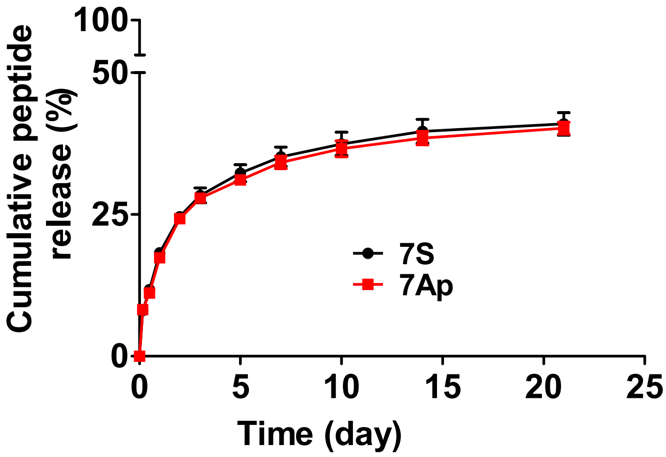

Figure S4. *In vitro* release of 7S and 7Ap peptide from the TEVGs. (n=6).

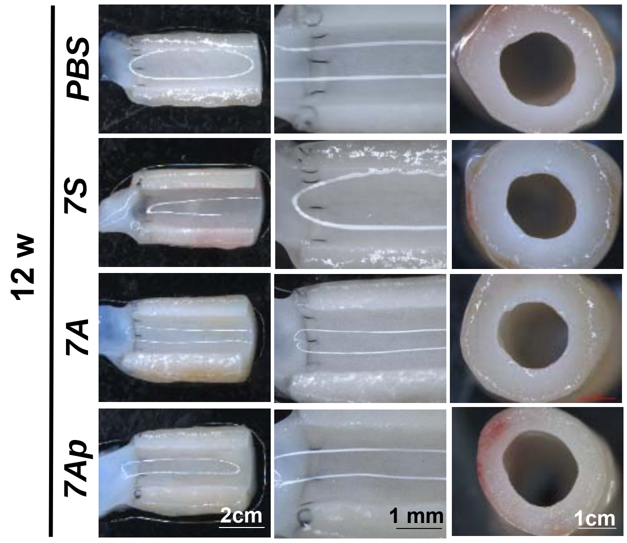

Figure S5. Stereomicroscopic images of explanted vascular grafts. The luminal surfaces of patent grafts were clean and free of thrombi at 12 weeks.

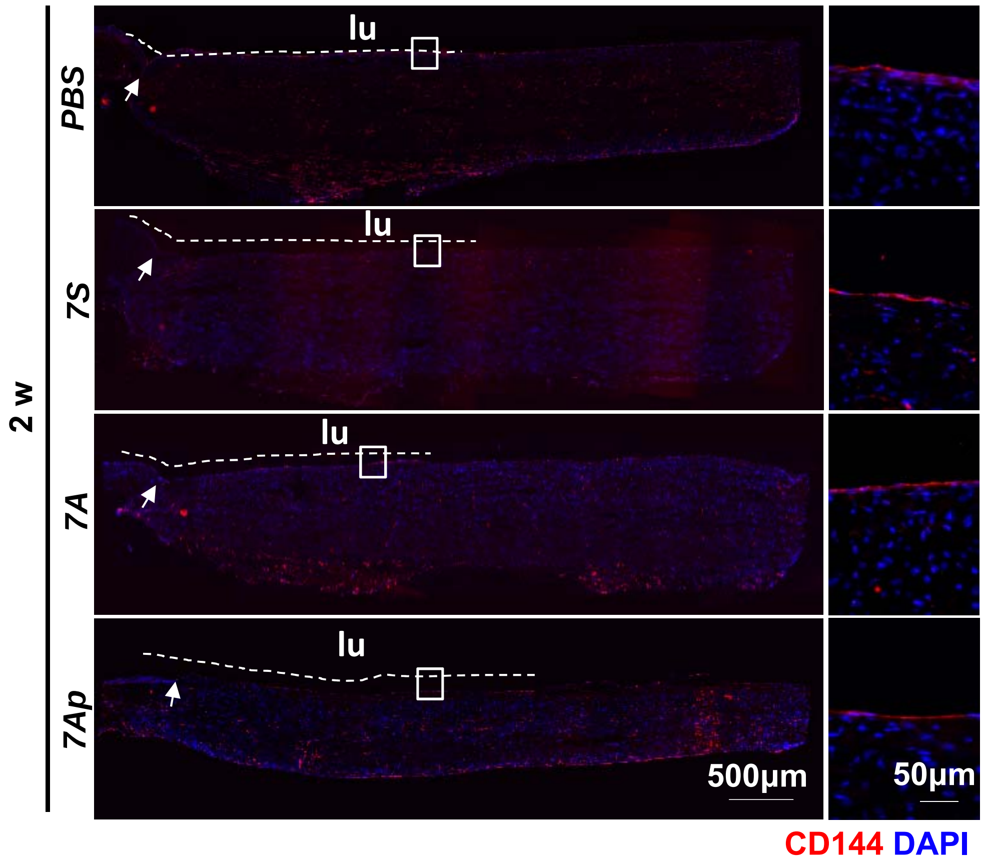

Figure S6. Endothelialization in vascular grafts after 2 weeks. The representative images of immunofluorescence staining of longitudinal section by CD144 antibody. Magnified image of the selected area was show in the right panel. The doted line represented endothelialized lumen, and the anastomotic site was indicated by the arrowhead. lu: lumen.

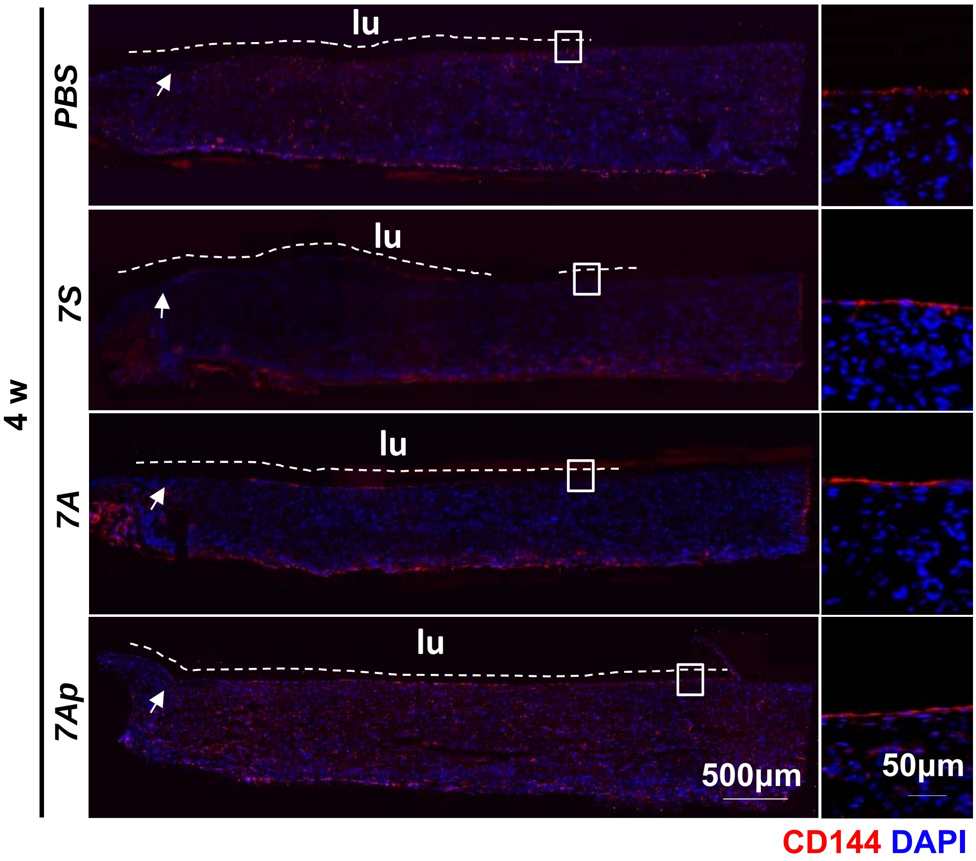

Figure S7. Endothelialization in vascular grafts after 4 weeks. The representative images of immunofluorescence staining of longitudinal section by CD144 antibody. Magnified image of the selected area was show in the right panel. The doted line represented endothelialized lumen, and the anastomotic site was indicated by the arrowhead. lu: lumen.

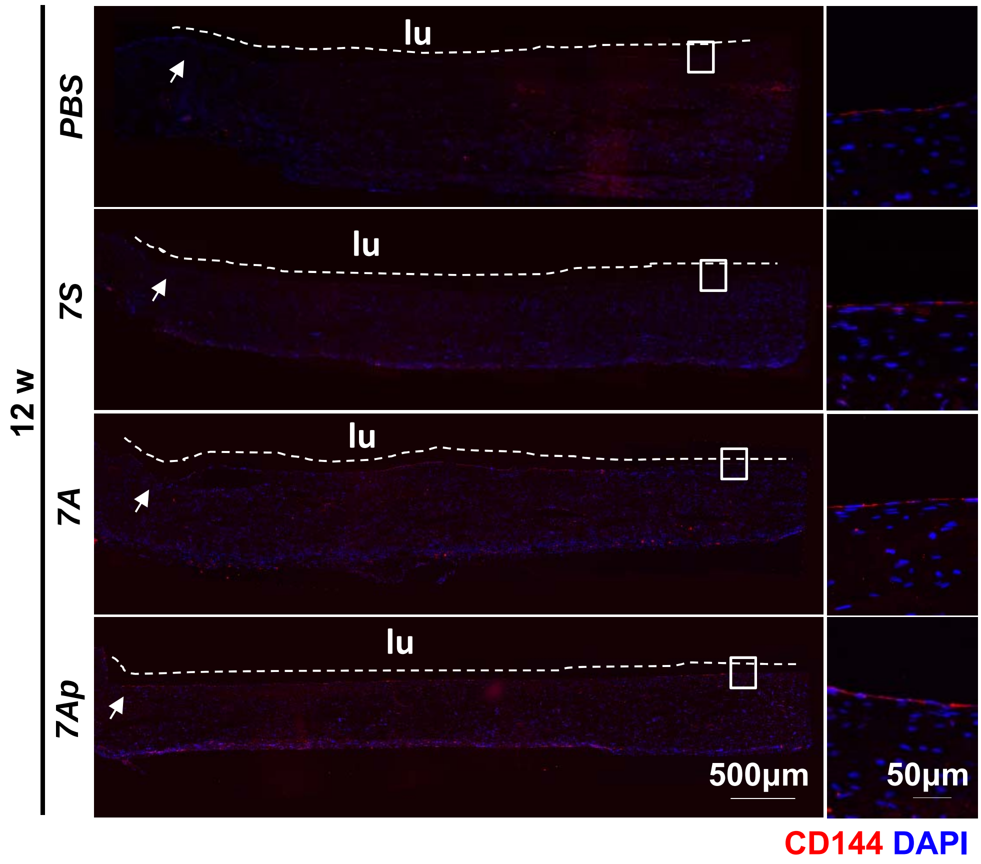

Figure S8. Endothelialization in vascular grafts after 12 weeks. The representative images of immunofluorescence staining of longitudinal section by CD144 antibody. Magnified image of the selected area was show in the right panel. The doted line represented endothelialized lumen, and the anastomotic site was indicated by the arrowhead. lu: lumen.

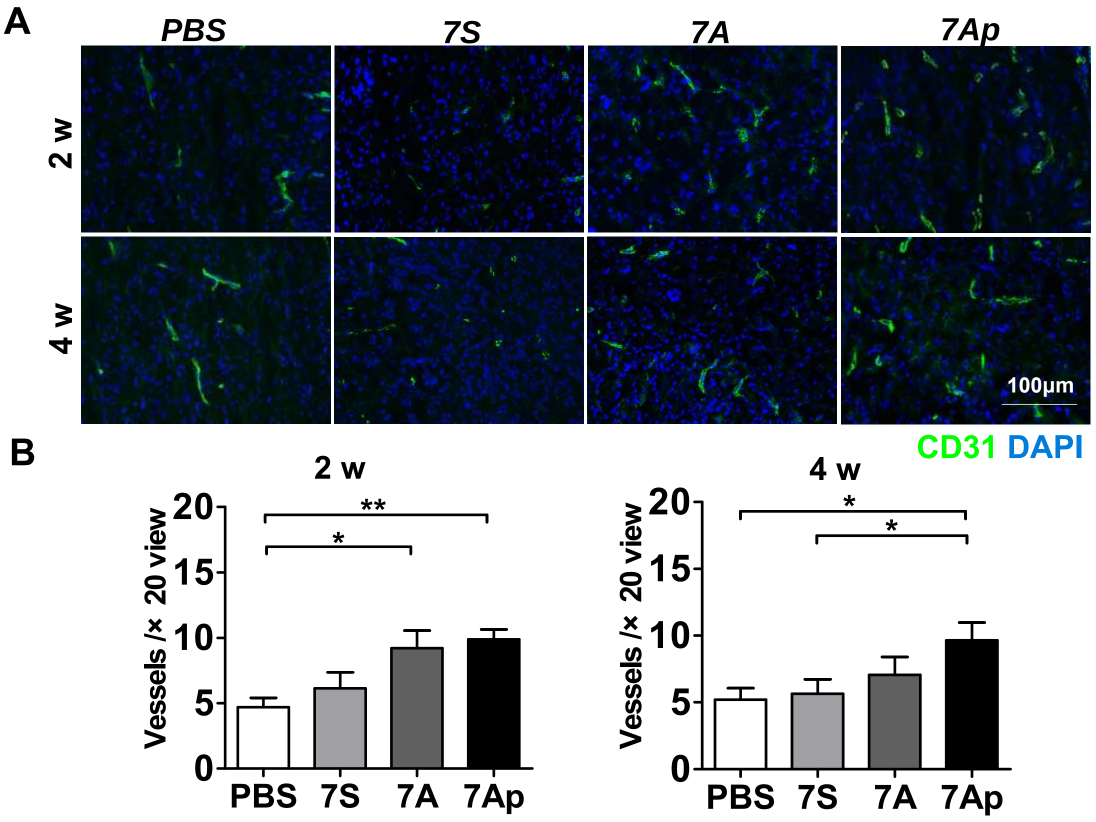

Figure S9. 7Ap increased capillary vessel formation in the PLTEVGs wall. Immunofluorescence staining was performed to detect the capillary vessels using anti-CD31 antibody (A), and corresponding quantification on vessel dendisty (B).

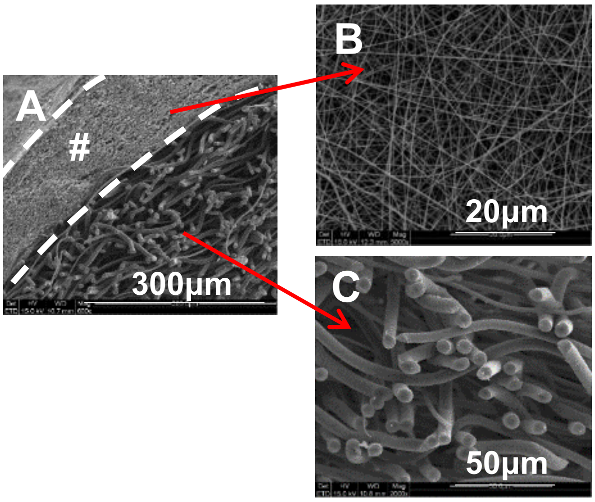

Measurement	Inner Layer	Outer Layer
**Fiber size/μm**	6.8±1.1	0.2±0.01
**Pore size/μm**	40.7±13.9	3.73±1.3
**Porosity**	81.5±2.1%	58.92±16.7%
**Thickness/μm**	326.8±25.5	238.6±38.1
**Total Wall Thickness/μm**	556.5±25.6	

Figure S10. Scanning electron microscopic (SEM) images show bi-layered structure with a low-porosity layer as outside barrier (#) (A). The fibrous structure of the two layers could be clearly observed from images at high magnification (B and C) and corresponding characterazation of the bi-layered graft.

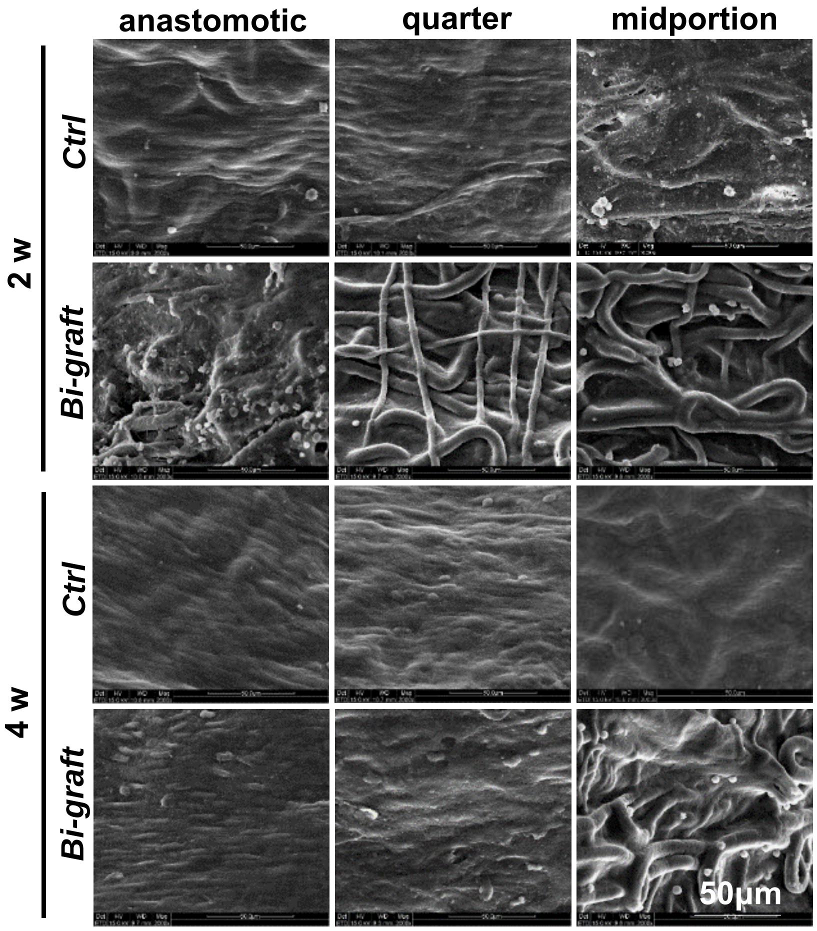

Figure 11. The endothelium coverage on the lumens of no-barrier (control) and outside-barrier (Bi-graft) vascular grafts at 2 and 4 weeks after implantation. Scanning electron microscopic (SEM) images of the lumens of no-barrier (control) and outside-barrier grafts at 2 and 4 weeks.

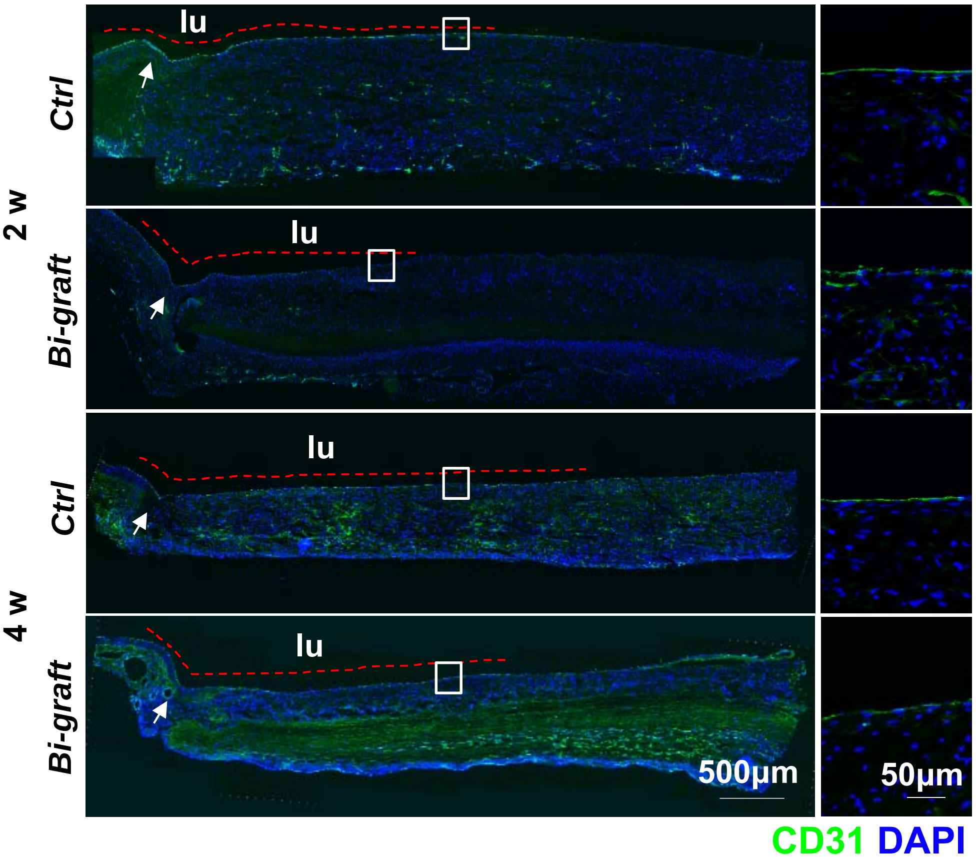

Figure S12. Endothelialization in the no-barrier (control) and outside-barrier (Bi-graft)vascular grafts after 2 and 4 weeks. The representative images of immunofluorescence staining of longitudinal section by CD31 antibodies, respectively. Magnified image of the selected area was show in the right panel. The doted line represented endothelialized lumen, and the anastomotic site was indicated by the arrowhead. lu: lumen.

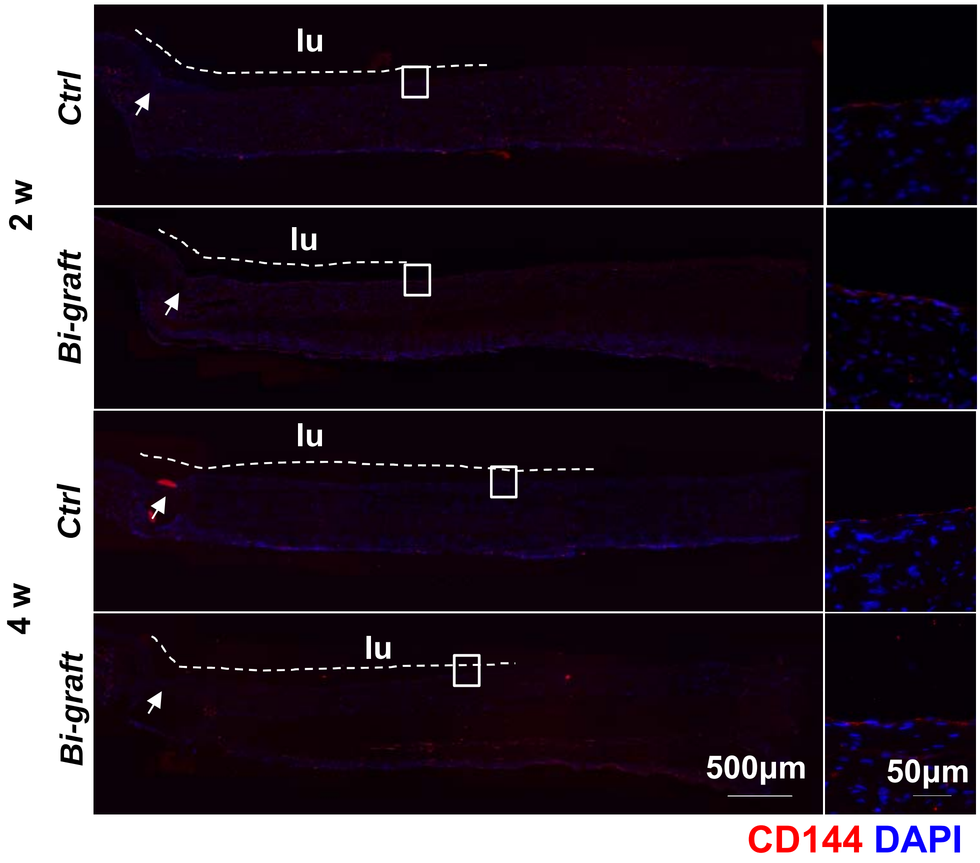

Figure S13. Endothelialization in the no-barrier (control) and outside-barrier (Bi-graft)vascular grafts after 2 and 4 weeks. The representative images of immunofluorescence staining of longitudinal section by CD144 antibodies, respectively. Magnified image of the selected area was show in the right panel. The doted line represented endothelialized lumen, and the anastomotic site was indicated by the arrowhead. lu: lumen.
